# Human Granulocytic Anaplasmosis, Japan

**DOI:** 10.3201/eid1902.120855

**Published:** 2013-02

**Authors:** Norio Ohashi, Gaowa, Wuritu, Fumihiko Kawamori, Dongxing Wu, Yuko Yoshikawa, Seizou Chiya, Kazutoshi Fukunaga, Toyohiko Funato, Masaaki Shiojiri, Hideki Nakajima, Yoshiji Hamauzu, Ai Takano, Hiroki Kawabata, Shuji Ando, Toshio Kishimoto

**Affiliations:** Author affiliations: University of Shizuoka and Global Center of Excellence Program, Shizuoka City, Japan (N. Ohashi, Gaowa, Wuritu, F. Kawamori, D. Wu, Y. Yoshikawa);; Kochi Institute of Health, Kochi City, Japan (S. Chiya, K. Fukunaga); Shizuoka Institute of Environment and Hygiene, Shizuoka City (F. Kawamori); Muroto Hospital, Muroto, Japan (T. Funato);; Kochi Prefectural Aki Hospital, Aki City, Japan (M. Shiojiri, H. Nakajima);; Chu-gei Clinic, Aki District, Japan (Y. Hamauzu); National Institute of Infectious Diseases, Tokyo, Japan (A. Takano, H. Kawabata, S. Ando);; Okayama Prefectural Institute for Environmental Science and Public Health, Okayama, Japan (T. Kishimoto)

**Keywords:** Anaplasma phagocytophilum, anaplasmosis, human granulocytic anaplasmosis, Rickettsia japonica, Orientia tsutsugamushi, rickettsiosis, p44/msp2, 16S rDNA, coinfection, Japan, spotted fever group rickettsia, ticks, bacteria

## Abstract

We retrospectively confirmed 2 cases of human *Anaplasma phagocytophilum* infection. Patient blood samples contained unique *p44/msp2* for the pathogen, and antibodies bound to *A. phagocytophilum* antigens propagated in THP-1 rather than HL60 cells. Unless both cell lines are used for serodiagnosis of rickettsiosis-like infections, cases of human granulocytic anaplasmosis could go undetected.

Japanese spotted fever (JSF) and scrub typhus, which are caused by infection with *Rickettsia japonica* and *Orientia tsutsugamushi*, respectively, are common rickettsioses in Japan (*1*). National surveillance (http://idsc.nih.go.jp/idwr/CDROM/Main.html [in Japanese]) indicates that JSF occurs frequently in central and western Japan and that scrub typhus is present throughout Japan, except in Hokkaido. In JSF- and scrub typhus–endemic areas, cases of non-JSP and non–scrub typhus disease with rickettsiosis-like fever have often been reported. And, human infection with *R. heilongjiangensis*, a spotted fever group (SFG) rickettsia, has been identified in Japan (*2*). Furthermore, *Anaplasma phagocytophilum* has been detected in *Ixodes persulcatus* and *I. ovatus* ticks, and *Ehrlichia chaffeensis* has been detected in deer (*3*–*6*). More recently, we identified *A. phagocytophilum* infection in ticks (*Haemaphysalis formosensis*, *H. longicornis*, *H. megaspinosa,* and *Amblyomma testudinarium*) from central and western Japan, the JSF-endemic areas of the country (*7*,*8*). We conducted this retrospective study to determine the cause of non-JSP and non–scrub typhus disease in 2 men in western Japan who had rickettsiosis-like fever.

## The Study

In 2002–2003 in Kochi Prefecture, western Japan, 2 men sought medical care for rickettsiosis-like signs and symptoms. Case-patient 1 (61 years old) sought care for fever (39.2°C), chills, and malaise 10 days after traveling to the mountains (day 0, the day of symptom onset). His physician prescribed cefdinir (300 mg/day). By day 3, signs and symptoms had not improved and an erythematous rash on his trunk had spread; the physician suspected infection with *R. japonica* or *O. tsutsugamushi*. The patient was hospitalized and intravenously administered minocycline (200 mg/day). Results (and reference values) for laboratory tests (day 3) follow: leukocytes, 5.8 × 10^9^ cells/L (3.5–9.2 × 10^9^ cells/L); thrombocytes, 225 × 10^9^ cells/L (155–365 × 109 cells/L); aspartate aminotransferase, 59 U/L (<38 U/L); alanine aminotransferase, 61 U/L (<36 U/L); and C-reactive protein, 12.1 mg/dL (<0.3 mg/dL).

Case-patient 2, a 73-year-old lumberjack, sought medical care for fever (39.2°C), headache, and malaise (day 0, the day of symptom onset). On day 4, a disseminated maculopapular rash was noticed, especially on the trunk and lower limbs; JSF or scrub typhus infection was suspected. The patient was hospitalized and intravenously administered minocycline (200 mg/day). Results for laboratory tests (day 4) follow: leukocytes, 6.4 × 10^9^ cells/L; aspartate aminotransferase, 100 U/L, alanine aminotransferase, 45 U/L; and C-reactive protein, 17.2 mg/dL.

In 2003, blood clots and serum samples from the 2 patients were transferred from Kochi Institute of Health to the University of Shizuoka, where they were stored at −20°C until a retrospective analysis could be performed. DNA was extracted from the blood clots, and nested PCR was performed, as described (*3*,*9*), to detect SFG rickettsiae 16S rDNA, *O. tsutsugamushi* 16S rDNA, *A. phagocytophilum p44/msp2*, and *Ehrlichia* spp. *p28/omp-1* (Table 1). To avoid DNA contamination, we performed PCR, electrophoresis, and cloning were performed in separate laboratories. As a negative control, nested PCR without DNA template samples was performed for each sample. PCR detected *A. phagocytophilum p44/msp2* multigenes in acute-phase blood clots from both case-patients, and SFG rickettsia 16S rDNA was amplified from a sample from case-patient 2 (Table 1).

**Table 1:** Results of PCR for select rickettsial organisms for 2 men with human granulocytic anaplasmosis, Kochi Prefecture, Japan*

Days after symptom onset†	Nested PCR result‡			
	SFG rickettsia 16S rDNA	*Orientia tsutsugamushi* 16S rDNA	*Anaplasma phagocytophilum p44/msp2*	*Ehrlichia* sp. *p28/omp-1*
Case-patient 1				
3	Negative	Negative	Positive	Negative
19	Negative	Negative	Negative	Negative
Case-patient 2				
4	Positive	Negative	Positive	Negative
11	NA	NA	NA	NA

*SFG, spotted fever group; NA, not available. †After in-hospital treatment with minocycline (200 mg/d), both case-patients improved clinically and were discharged on days 20 and 12, respectively, after symptom onset. ‡Before being used in PCR, blood clots from the patients were homogenized by using BioMasher (Nippi Inc., Tokyo, Japan) and treated overnight with 100 U of streptokinase (WAKO Pure Chemical Industries Ltd, Osaka, Japan). DNA then was extracted by using the QIAamp DNA Mini Kit (QIAGEN, Valencia, CA, USA). Multiplex nested first-step PCR for SFG rickettsiae and *O. tsutsugamushi* was performed by using the following primers: RO-1F (5'-CCGTAAACGATGAGTGCTAGA-3') and RO-R1 (5'-CCGAGAACGTATTCACCGC-3'). Multiplex nested second-step PCR for SFG rickettsiae 16S rDNA was performed by using the following primers: R-2F (5'-GAAGATTCTCTTTCGGTTTCGC-3') and R-2R (5'-GTCTTGCTTCCCTCTGTAAAC-3'). Multiplex nested second-step PCR for *O. tsutsugamushi* 16S rDNA was performed by using the following primers: O-2F (5'-GACATGGTAGTCGCGAAAAATG-3') and O-2R (5'-TGCAATCCGAACTGAGATACC-3'). *A. phagocytophilum p44/msp2* was amplified by using primers p3726, p4257, p3761, and p4183, and *Ehrlichia* spp. *p28/omp-1* was amplified by using primers conP28-F1, conP28-R1, conP28-F2, and conP28-R2, as described (*3*,*9*).

**Figure 1:**
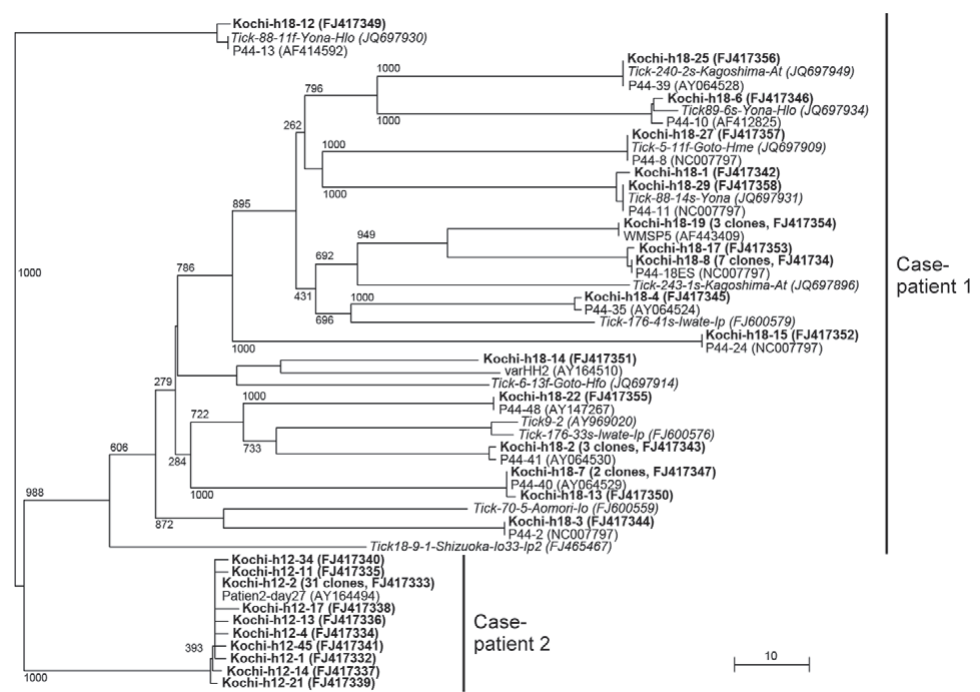
Phylogenetic analysis of *Anaplasma phagocytophilum p44/msp2* multigenes detected in blood from 2 men in Kochi Prefecture, Japan. Each *p44/msp2* PCR product was cloned (TA Cloning Kit; Life Technologies, Grand Island, NY, USA) into the PCR2.1 vector, after which recombinant clones were randomly selected and the DNA inserts were sequenced. The tree was constructed on the basis of the 117–133 aa sequences of the *p44/msp2* genes by using the neighbor-joining method. The closest relatives to sequences for the 2 case-patients are included in the tree. Those sequences have been published in GenBank: patient2-day27 (obtained from a US patient); P44-2, P44-8, P44-10, P44-11, P44-13, P44-18E, P44-28, P44-35, P44-39, P44-40, P44-41, P44-48, varHH2, and WMSP5 are from human isolates; and 44-kDa outer membrane proteins are from ticks collected in Japan. **Boldface** font indicates the 28 *p44/msp2* genes from case-patient 1 and the 40 from case-patient 2. Numbers on the tree indicate bootstrap values for branch points. Scale bar indicates the percent of sequence divergence. Data in parentheses indicate the number of *p44/msp2* clones with identical sequences and the sequence accession numbers.

Amplicons of *p44/msp2* were subjected to TA cloning (TA Cloning Kit; Life Technologies, Grand Island, NY, USA), and randomly selected recombinant clones were sequenced and analyzed phylogenetically (Figure 1). A total of 28 *p44/msp2* clones from case-patient 1 shared 27.5%–100% similarity with each other and were widely dispersed in the tree. The 40 clone sequences from case-patient 2 shared 97.5%–100% similarity with each other and grouped into a single cluster. Using Blast (http://blast.ncbi.nlm.nih.gov), we compared the sequences with those in GenBank; 27 previously identified *p44/msp2* variants from human isolates and ticks collected in Japan were identified as the closest relatives to *p44/msp2* cloned from the 2 patients. We included the 27 variants in the tree; however, some were widely separated from the related clones (Figure 1). For case-patient 2, the 389-bp sequence of the 16S rDNA amplicon (determined by direct sequencing) was 100% identical to that of *R. japonica* YH (GenBank accession no. AP011533).

**Figure 2:**
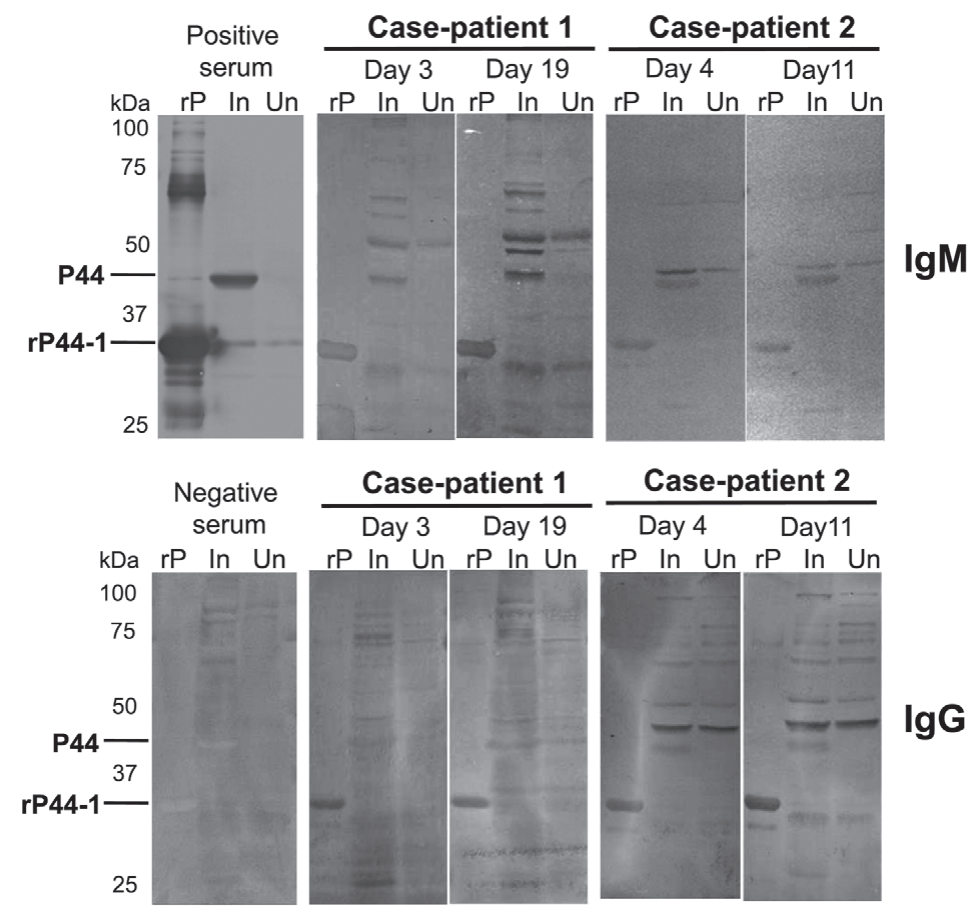
Western blot analyses, using recombinant P44-1 protein (rP44-1) and *Anaplasma phagocytophilum*–infected THP-1 cells as antigens, of serum samples from 2 men, case-patients 1 and 2, who had *A. phagocytophilum* infection, Kochi Prefecture, Japan. The *Escherichia coli*, which produced rP44-1, was kindly provided by Yasuko Rikihisa (Ohio State University, Columbus, OH, USA). The rP44-1 and the rabbit hyperimmune serum (positive control serum) were prepared as described (*11*,*12*). Results for a negative control (human serum sample) are included. The primary human serum samples tested were diluted 200- to 400-fold; rabbit serum sample (positive control) was diluted 2,000-fold. The goat antihuman IgG and IgM alkaline phosphatase conjugates (Life Technologies, Grand Island, NY, USA) were used as secondary antibodies. Days represent days after symptom onset. rP, rP44-1 antigen; In, infected THP-1; Un, uninfected THP-1 cells.

Serologic evidence of infection was demonstrated by using indirect immunofluorescence assay (IFA) and Western blot analysis as described (*10*,*11*). In IFAs, IgM and/or IgG from serum samples from the case-patients reacted with *A. phagocytophilum* cultured in THP-1 rather than HL60 cells, and seroconversion was stronger in convalescent-phase serum samples (Table 2). IgG titers against *R. japonica* were also higher in convalescent-phase samples from case-patient 2. Western blot analysis further confirmed the specific reaction to the 44-kDa outer membrane proteins (P44s) of *A. phagocytophilum* cultured in THP-1 cells and/or to the recombinant P44-1 (rP44-1) in serum samples (Figure 2, Table 2). However, using the same serum samples, we could not detect P44 antigens of *A. phagocytophilum* propagated in HL60 cells (data not shown), supporting the IFA result.

**Table 2:** Detection of IgM and IgG in serum samples from 2 men with human granulocytic anaplasmosis, Kochi Prefecture, Japan*

Days after symptom onset	Antibody titers, IgM/IgG†			
	*R. japonica*, cultured in L929 cells‡	*O. tsutsugamushi*, cultured in L929 cells§	*Anaplasma phagocytophilum*, propagated in	
			HL60 cells	THP-1 cells
Case-patient 1				
3	<20/<20	<20/<20	<20/<20	80/<20
19	<20/<20	<20/<20	<20/<20	320/80
Case-patient 2				
4	<20/<20	<20/<20	20/<20	40/40
11	<20/320	<20/<20	<20/<20	160/80

*All Western blot testing using recombinant P44-1 antigen detected IgM and IgG; the antigen reacted with all sera tested, as shown in Figure 2.  †Determined by using indirect immunofluorescence assay. ‡*Rickettsia japonica* strain YH. §*Orientia tsutsugamushi* strains Gilliam, Karp, Kato, and Kawasaki.

In central and western Japan, most cases of tickborne infectious and febrile disease have been reported as JSF (*1*,*13*), and *R. japonica* has been frequently detected in ixodid ticks in these areas. We found *A. phagocytophilum* infection in several species of ticks, and at least 3 species (*H. formosensis*, *H. longicornis*, and *I. ovatus*) seem to be associated with *R. japonica* and *A. phagocytophilum* (*7*,*8*). National surveillance during 1999–2010, showed that JSF was endemic in Kochi Prefecture during 1999–2004. More recently, JSF-endemic areas are Mie, Kagoshima, Wakayama, and Kumamoto Prefectures rather than Kochi Prefecture. Our survey demonstrating the presence of *A. phagocytophilum*–infected ticks in Mie and Kagoshima Prefectures (*8*) indicates that there is a risk for dual infection with *R. japonica* and *A. phagocytophilum* in JSF-endemic areas of Japan.

*A. phagocytophilum* cultured in HL60 cells is generally used as a source of antigen for serodiagnosis of human anaplasmosis. Our findings show, however, that titers of antibody against *A. phagocytophilum* propagated in THP-1 cells were higher than those propagated in HL60 cells. We further analyzed the transcription of *p44/msp2* multigenes encoding P44 repertoires (major antigens of *A. phagocytophilum*) in infected HL60 and THP-1 cells by using reverse transcription PCR followed by TA cloning as described (*7*). The analyses showed that a transcript from the *p44-60* gene and another from the *p44-47* gene (75% and 25% of transcripts tested, respectively) were dominantly expressed in *A. phagocytophilum* propagated in THP-1 cells but not in HL60 cells; several transcript species other than *p44-60* and *p44-47* of *p44/msp2* multigenes were expressed in *A. phagocytophilum* propagated in HL60 cells (data not shown). A previous proteomic study supported the variety of P44 repertoires produced by *A. phagocytophilum* in HL60 cells (*14*). The difference of *p44/msp2* expression between HL60 and THP-1 cell cultures may reflect the discrepancy of antibody titers obtained by IFAs. Furthermore, in IFAs using infected THP-1 antigens, IgM titers tended to be higher than IgG titers, even in convalescent-phase serum samples. These patients probably produced IgG reactive with P44 species other than P44-60 and P44-47 that were dominantly expressed in *A. phagocytophilum* propagated in THP-1 cells; Western blot analysis showed that IgG in patients strongly bound to recombinant P44-1 rather than P44s (probably including P44-60 and P44-47) of *A. phagocytophilum* propagated in THP-1 cells. Thus, cases of human anaplasmosis could go undiagnosed if only infected HL60 cells, and not THP-1 cells, are used as antigen for serodiagnosis of rickettsiosis-like infections, as is currently done when using IFAs.

## Conclusions

We documented 2 cases of human granulocytic anaplasmosis in Japan, 1 with and 1 without JSF coinfection. To avoid misdiagnosing cases of human anaplasmosis, we recommend that *A. phagocytophilum* propagated in THP-1 and in HL60 cells be used as antigens for the serodiagnosis of rickettsiosis-like infections.
